# The impact of bZIP Atf1ortholog global regulators in fungi

**DOI:** 10.1007/s00253-021-11431-7

**Published:** 2021-07-24

**Authors:** Éva Leiter, Tamás Emri, Klaudia Pákozdi, László Hornok, István Pócsi

**Affiliations:** 1 Department of Molecular Biotechnology and Microbiology, Institute of Biotechnology, University of Debrecen, P.O. Box 63, Debrecen, H-4010 Hungary; 2 Hungarian University of Agriculture and Life Sciences, Gödöllő, Hungary

**Keywords:** bZIP-type transcription factor, Vegetative growth, Development, Environmental stress regulatory network, Secondary metabolism, Virulence

## Abstract

**Abstract:**

Regulation of signal transduction pathways is crucial for the maintenance of cellular homeostasis and organismal development in fungi. Transcription factors are key elements of this regulatory network. The basic-region leucine zipper (bZIP) domain of the bZIP-type transcription factors is responsible for DNA binding while their leucine zipper structural motifs are suitable for dimerization with each other facilitiating the formation of homodimeric or heterodimeric bZIP proteins. This review highlights recent knowledge on the function of fungal orthologs of the *Schizosaccharomyces pombe* Atf1, *Aspergillus nidulans* AtfA, and *Fusarium verticillioides* FvAtfA, bZIP-type transcription factors with a special focus on pathogenic species. We demonstrate that fungal Atf1-AtfA-FvAtfA orthologs play an important role in vegetative growth, sexual and asexual development, stress response, secondary metabolite production, and virulence both in human pathogens, including *Aspergillus fumigatus*, *Mucor circinelloides, Penicillium marneffei*, and *Cryptococcus neoformans* and plant pathogens, like *Fusarium* ssp., *Magnaporthe oryzae*, *Claviceps purpurea*, *Botrytis cinerea*, and *Verticillium dahliae.*

**Key points:**

• *Atf1 orthologs play crucial role in the growth and development of fungi*.

• *Atf1 orthologs orchestrate environmental stress response of fungi*.

• *Secondary metabolite production and virulence are coordinated by Atf1 orthologs*.

## Introduction

Basic-region leucine zipper (bZIP)-type transcription factor proteins can form either homodimers or heterodimers with other bZIP-type transcription factors. Their DNA binding basic motifs interact with cAMP response elements (CREs) present in a number of promoters and, as a result, they control and organize a wide array of cellular processes. In fact, bZIP-type transcription factors represent one of the most ancient transcription factor families evolved from a single eukaryotic gene. They contribute to a complex regulatory network, which plays a pivotal role in differentiation and maintenance of cells as well as orchestration of stress responses in multicellular eukaryotes (Jindrich and Degnan 2016). In plants, bZIP transcription factors (e.g., AtbZIP1 in *Arabidopsis thaliana* and BZI-1 in *Nicotiana tabacum*) are involved in development (e.g., seed induction), as well as in responses to both abiotic (e.g*.*, drought, cold, salinity, high temperature) and biotic (i.e., herbivores, insects, and microbial pathogens) stresses (Alves et al. 2013). In humans, the bZIP-type activating transcription factor ATF2 (activating transcription factor 2) coordinates cellular homeostasis and normal organismal development, and is also involved in pathological processes (Watson et al. 2017). ATF2 regulates the expression of key genes involved in inflammatory processes, cell cycle, glycosylation, and responses to amino acid limitations (Watson et al. 2017). Unfortunately, various disorders are also associated with altered ATF2 expression and/or its phosphorylation, and ATF2-coupled pathological processes may affect, among others the kidneys, the nervous system, and the pancreas and may contribute to the development of different types of cancer (Watson et al. 2017).

The orthologs of ATF2 have also been identified in the genomes of fungi starting with Atf1, the first ATF2 orthologous protein described in *Schizosaccharomyces pombe*. Atf1 is involved in stress response, sexual development (Takeda et al. 1995), and meiotic hot spot activation (Kon et al. 1998; Hirota et al. 2007) of the fission yeast. It easily forms heterodimers with Pcr1, another bZIP-type transcription factor, but the significance of this process seems to be unequal in stress response and mating with preference for the latter in fission yeast (Kon et al. 1998; Sansó et al. 2008). According to DNA microarray-based transcriptome analyses performed in *S. pombe*, a core environmental stress response (CESR) mounted to oxidative, osmotic, heavy metal, alkylating, and heat stresses exists in this yeast. CESR is controlled mainly by Sty1 (Spc1) mitogen-activeted protein kinase (MAPK) and, to some extent, by Atf1 acting downstream of Sty1 (Gasch 2007; Chen et al. 2003). It is worth mentioning that there are stress response genes, whose regulation seems to be Atf1 independent in *S. pombe* but their expression is controlled predominantly via mRNA turnover (Marguerat et al. 2014).

Several studies demonstrated that Atf1 expression is controlled both transcriptionally and posttranscriptionally in stress-exposed *S. pombe*. Leong et al. (2014) described a non-coding RNA locus (*SPNCRNA.1164*) that positively controls both *atf1* expression and oxidative stress response. In the fission yeast, expression levels of the oxidative stress response bZIP transcription factors, Atf1 and Pcr1, increase under H_2_O_2_ stress contributing to the successful adaptation of this fungus to oxidative stress. Interestingly, *atf1* and *pcr1* mRNAs contain a biased number of AAA versus AAG codons, both coding for lysine. Since the RNA polymerase II-interacting elongation complex also has an impact on the modification of some tRNAs (e.g., at the uridine wobble nucleosides), it may modulate translation efficiency in this way as well. Not surprisingly, when the histone acetyl transferase Sin3/Elp3 localized in the Elongation complex was impaired, the AAA codons were insufficiently translated resulting in reduced *atf1* and *pcr1* expressions and as a consequence oxidative stress-sensitive phenotypes (Fernández-Vázquez et al. 2013).

Atf1 itself controls the expression of stress-responsive genes in different manners. For example, it recruits Set1, the catalytic subunit of the highly conserved Set1C/COMPASS complex responsible for histone H3K4 methylation (H3K4me) and sets the stress-responsive and developmental genes in “poised” off state to facilitate their rapid expression, if necessary (Lorenz et al. 2014). The Sty1 (Spc1) MAPK-activated Atf1-Pcr1 bZIP heterodimer binds to oxidative stress-responsive RNAs containing the *M26* sequence motif (5′-UGACGU-3′ or 5′-ACGUCA-3′) in addition to CRE-like DNA sites (5′-ATGACGT-3′) and, in this manner, facilitates the stress-induced decay of these ribonucleic acids (Gao et al. 2013). It is worth mentioning that the 5′-ATGACGT-3′ sequence is also essential for *M26* meiotic hot spot activation in the *ade6* gene (Kon et al. 1998; Steiner et al. 2002; Hirota et al. 2007).

The genome of *Saccharomyces cerevisiae* also encompasses Atf-like proteins (Takeda et al. 1995), but fungi summarized in this review are closer to *S. pombe* if we consider their stress response system including the Atf1 ortholog regulatory network (Miskei et al. 2009).

Since the first pioneering studies published on the versatile developmental and physiological functions of the fission yeast’s Atf1, mycologists have successfully elucidated the outstanding role played by Atf1 orthologs in other fungal taxa including the saprophytic model organisms, *Aspergillus nidulans* and *Neurospora crassa*, the opportunistic human parasitic fungus *Aspergillus fumigatus*, and plant pathonenic fungi including *Fusarium graminearum* and *Fusarium verticillioides*. In this review, we aim to focus on the diverse and complex developmental and physiological functions of the Atf1-type transcription factors in the Kingdom of Fungi.

## Atf1-AtfA-FvAtfA orthologs regulate vegetative growth and development

**Fig. 1:**
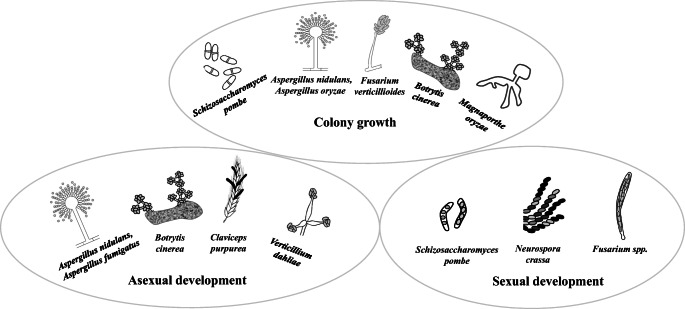
**A**tf1-AtfA-FvAtfA orthologs are involved in the maintenance of vegetative gowth as well as in asexual and sexual developments of fungi. Schematic vegetative, asexual, and sexual morphological forms of fungi with successful functional characterization of Atf1-AtfA-FvAtfA orthologs are presented

The versatile involvements of Atf1 (*S. pombe*)*-*AtfA (*A. nidulans*)-FvAtfA (*F. verticillioides*) orthologs in maintenance of vegetative growth and regulation of asexual and sexual development of fungi are summarized in Figure 1.

In. *S. pombe*, the lack of *atf1* resulted in reduced colony size at 30 °C while no colony growth was observed at lower temparetures (Takeda et al. 1995). The *Δatf1* strain failed to mate with otherwise compatible partners indicating the crucial role of this transcription factor in the sexual development of *S. pombe* (Takeda et al. 1995; Shiozaki and Russell 1996).

Orthologs of *S. pombe atf1* are of outstanding importance in the coordination of growth as well as the formation of asexual and sexual spores in filamentous fungi including *A. nidulans* and *N. crassa* (Hagiwara et al. 2016; Colot et al. 2006). In *A. nidulans*, deletion of *atfA* affected vegetative growth and conidiospore formation (Balázs et al. 2010; Emri et al. 2015). The lack of *atfA* decreased the expression of a number of genes involved in conidiophore development as revealed by microarray-based transcriptome analysis (Emri et al. 2015; Antal et al. 2020) confirming the observation that *ΔatfA* mutants showed moderate growth and produced less conidia than the control strains (Balázs et al. 2010; Emri et al. 2015). In *N. crassa*, the other widely used model filamentous fungus and ascospores of the *atf1* ortholog *asl-1* gene deletion mutant were unable to germinate indicating the pivotal function of Asl-1 in completing the sexual cycle (Colot et al. 2006).

In the koji mold, *Aspergillus oryzae* aerial hyphal surface of the *ΔatfA* colonies was thinner than that of the control strain suggesting the role of AtfA in aerial hyphal elongation (Sakamoto et al. 2009). Germination ratio of conidia of *ΔatfA* was reduced compared to the control strain as a result of decreased glutamate level in conidiospores of *ΔatfA* (Sakamoto et al. 2009). Deletion of *atfA* brought about the loss of viability of conidiospores after 2-week incubation at 4 °C in *A. fumigatus* (Hagiwara et al. 2014). In the *ΔatfA* mutant, an unusual metabolic activity was manifested in increased respiration and premature germination even in the absence of nutrients leading to an abnormal release from dormancy (Hagiwara et al. 2016). According to a comparative transcriptomic analysis of *A. oryzae*, *Aspergillus niger* (a black mold used in industrial scale citric acid production), and *A. fumigatus atfA* contributed to conidial dormancy by negatively regulating genes associated with spore germination. Compared with the *A. fumigatus* conidia associated genes (CAGs), the common *Aspergillus* CAGs contained a higher percentage (64%) of AtfA-dependent genes, indicating that AtfA is important in conidial maturation. All these results support the assumption that AtfA orthologs play important functions in conidiogenesis, spore maturation, spore germination, and the maintenance of viability and stress tolerance during spore dormancy in aspergilli (Hagiwara et al. 2016).

Considering other opportunistic human pathogens, the genome of *Mucor circinelloides* harbors two *S. pombe atf1* orthologous genes, *atf1* and *atf2* (Pérez-Arques et al. 2019). Both genes are important elements of the virulence of *M. circinelloides* by contributing to the growth and development of the fungus during macrophage interaction as well as to the germination of spores inside the macrophages (Pérez-Arques et al. 2019). Interestingly, in another human pathogenic fungus, *Penicillium marneffei*, deletion of *atfA* caused no harm of vegetative growth or asexual development (Nimmanee et al. 2014).

Large sets of phenotypic data are also available for *atf1*-*atfA* orthologous gene deletion strains of various plant pathogenic fungi. Developmental phenotypes were observed in fusaria, e.g., deletion of *Fgatf1* led to delayed perithecia formation with viable ascospores in *F. graminearum* (Nguyen et al. 2013). In another study with *F. graminearum*, the role of *Fgatf1* in sexual reproduction was also confirmed, as the deletion mutant showed delayed ascospore release (Jiang et al. 2015). In the *ΔFgatf1 ΔFgSKN7* (*SKN7* encodes a nuclear response regulator and transcription factor in baker’s yeast) double deletion mutant, asci accomodated eight small, single-celled ascospores instead of the typical four-celled ascospores (Jiang et al. 2015). The same double gene deletion also disturbed the asexual sporulation of *F. graminearum* resulting in smaller conidia in comparison to the wild-type strain. In *Fusarium oxysporum* f. sp. *cubense*, *Foatf1* had no role in regulation of mycelial growth, but its deletion led to the formation of smaller conidia with dramatically reduced germination rates in comparison to the wild-type strain (Qi et al. 2013). In *F. verticillioides*, the *ΔFvatfA* mutant exhibited decreased vegetative growth and reduced conidium production; furthermore, conidia of the mutant were significantly smaller than that of the wild type. Unlike to the aspergilli, viability of the mutant conidia was similar to that of the wild type (Szabó et al. 2020). Reduced vegetative growth was also described in the *MoAtf1* deletion mutant of *Magnaporthe oryzae* with sparse aerial hyphae emerging on complex medium (Guo et al. 2010). Developmental defects of the *ΔMoAtf1* mutant also arose in the formation of infectious hyphae since appressoria showed impaired phenotype with swollen and dark colored shortened hyphae (Guo et al. 2010).

Interestingly, the formation of sclerotia was damaged in the *Δcptf1* (*cptf1* is orthologous to *atf1*) mutant of the ergot fungus *Claviceps purpurea* (Nathues et al. 2004). In the grey mold fungus, *Botrytis cinerea*, the *Δbcatf1* mutant showed defects in both conidia production and sclerotia formation demonstrating that BcAtf1 was involved in the asexual development of this pathogenic fungus (Temme et al. 2012). BcAtf1 also plays an important role in the vegetative growth of hyphae because the lack of the *bcatf1* gene stimulated the robust growth of aerial hyphae manifesting in a fluffy phenotype (Temme et al. 2012), which could also be observed during plant surface colonization. As showed by microarray data, expression of genes with functions in cell wall biogenesis and plant cell wall degradation was disturbed. Nevertheless, the germination rates of conidia of the wild-type and mutant strains were similar (Temme et al. 2012). In *Verticillium dahliae*, deletion of the ATF ortholog *VDAG_08676* resulted in significantly less conidia without any impact on microsclerotia formation (Fang et al. 2017).

## Atf1-AtfA-FvAtfA orthologs coordinate a broad spectrum of stress responses

**Fig. 2:**
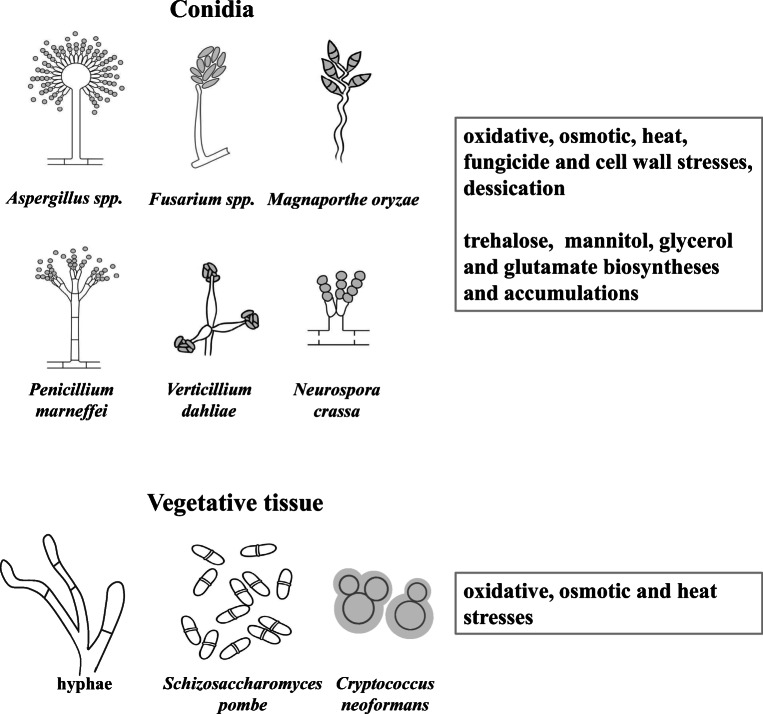
Atf1-AtfA-FvAtfA orthologs coordinate environmental stress response of fungi. Typical morphological forms of fungi and major stress responses governed by Atf1-AtfA-FvAtfA orthologs are presented

bZIP-type transcription factors are crucially important in the orchestration of the environmental stress response of fungi (Chen et al. 2003; Emri et al. 2015; Rodrigues-Pousada et al. 2010) (Figure 2, Table 1). Not surprisingly, the lack of *atf1* resulted in osmotic stress sensitivity when *S. pombe* was exposed to high osmolarity stress in the presence of KCl (Shiozaki and Russell 1996). The survival rate of the *Δatf1* mutant after heat and oxidative stresses was also significantly lower than that of the wild-type strain (Sansó et al. 2008).

**Table 1:** Atf1-AtfA-FvAtfA orthologs involved in the stress responses of fungal species

Species	Orthologs	Stress	Reference
*S. pombe*	Atf1	Osmotic (KCl) Heat (45 °C) Oxidative (H_2_O_2_)	Shiozaki and Russell 1996; Sansó et al. 2008
*A. nidulans*	AtfA	Oxidative (*t*BOOH, MSB, H_2_O_2_) Heat (50 °C) Fungicide (fludioxonil)	Balázs et al. 2010; Hagiwara et al. 2008; Lara-Rojas et al. 2011; Emri et al. 2015; Hagiwara et al. 2009
*N. crassa*	Atf-1/Asl-1	Osmotic (NaCl) Fungicide (fludioxonil)	Yamashita et al. 2008; Colot et al. 2006
*A. fumigatus*	AtfA	Cold (4 °C) Dessication Heat Oxidative (*t*BOOH, MSB, H_2_O_2_, paraquat) Osmotic (sorbitol) Cell wall (Congo Red and calcofluor white) Fungicide (fluodioxonil and caspofungin)	Hagiwara et al. 2014; Valero et al. 2020; Silva et al. 2016, 2021
*P. marneffei*	AtfA	Oxidative (*t*BOOH)	Nimmanee et al. 2014
*A. oryzae*	AtfA	Oxidative (H_2_O_2_)	Sakamoto et al. 2009
*F. graminearum*	FgAtf1	Oxidative (H_2_O_2_) Osmotic (NaCl, KCl, and sorbitol) Cell wall (Congo Red)	Jiang et al. 2015; Nguyen et al. 2013
*F. verticillioides*	FvAtfA	Oxidative (H_2_O_2_, MSB) Cell wall (Congo Red)	Szabó et al. 2020
*M. oryzae*	MoAtf1	Oxidative (H_2_O_2_)	Guo et al. 2010

In functional genomic studies in filamentous fungi, the ascomycetous fungi *A. nidulans* and *N. crassa* are the most favored model organisms. In *A. nidulans*, deletion of *atfA* negatively affected the oxidative stress (e.g., *t*BOOH-*tert-*butyl-hidroperoxide and MSB-menadione sodium bisulfite) tolerance of vegetative structures (Balázs et al. 2010; Hagiwara et al. 2008; Lara-Rojas et al. 2011; Emri et al. 2015). In addition, conidia of the *ΔatfA* mutant showed increased sensitivity to oxidative, heat, osmotic, and fungicide stresses (Hagiwara et al. 2008, 2009). Hagiwara et al. (2008) and Balázs et al. (2010) also observed dramatic decrease in viability after long-term storage at 4 °C in the *ΔatfA* mutant. Furthermore, conidia of the gene deletion strain showed increased heat sensitivity at 50 °C after 10- and 20-min incubation (Hagiwara et al. 2008).

In *N. crassa*, *atf-1* is important in the response to osmotic stress and fludioxonil. Without any observable growth difference between the wild type strain and the *Δatf-1* mutant, several genes involved in osmotic and fludioxonil stresses, i.e., *cat-1* (conidia-specific catalase gene), *bli-3* (bluelight-inducible gene), and *ccg-1* (clock-controlled gene), were downregulated in the gene deletion strain (Yamashita et al. 2008). In addition, catalase (CAT1) enzyme activity was abolished in the conidia of the *Δatf-1* mutant indicating that ATF-1 controls *cat-1* gene expression during both conidiation and osmoadaptation. Nevertheless, catalase-peroxidase (CAT2) activities were similar to activities observed in the wild-type strain (Yamashita et al. 2008).

In *A. oryzae*, conidia of the *ΔatfA* mutant were hypersensitive to H_2_O_2_ resulting in inhibition of conidial germination (Sakamoto et al. 2009). According to microarray analysis of global gene expression changes, a number of genes involved in oxidative stress responses were repressed in the *ΔatfA* strain, including catalase (including *catA* and a *S. cerevisiae CTA1* homolog), trehalose-6-phosphate synthase, thioredoxin, glutathione S-transferase, γ-glutamylcystein synthase, and heat shock protein genes (Sakamoto et al. 2009). Some stress-responsive genes were also under the control of another bZIP-type transcription factor, AtfB, but AtfA influenced the expression of a lot more genes (Sakamoto et al. 2009) similarly to the asymmetry observed within the Atf1-Pcr1 transcription factor pair in *S. pombe*. In *A. oryzae*, the bZIP transcription factors tested (as indicated in parentheses) affected the trehalose and mannitol content of conidiospores (AtfA and AtfB), the biosynthesis of glycerol during conidial germination (AtfA), and glutamate accumulation in the spores (AtfA) (Sakamoto et al. 2009). Deletion of *atfB* also increased the oxidative stress sensitivity of *A. oryzae* conidia but less markedly than observed after the elimination of *atfA* (Sakamoto et al. 2008).

Stress-sensitive phenotypes have also been described for *A. fumigatus* (Hagiwara et al. 2014). After 14-day incubation at 4 °C or dessication under vacuum, conidiospores of the *ΔatfA* mutant were unable to germinate underlying the central role of *atfA* in the maintenance of conidial viability. Regulation of some important stress-related genes was AtfA dependent in *A. fumigatus* conidia, including *catA* (catalase A), *dprA* (dehydrin-like protein), *scf1* (heat shock protein), and *conJ* (conidiation-specific Con10 protein), and the mutant spores contained less trehalose (Hagiwara et al. 2014). In further studies by Silva et al. (2016, 2021), the *ΔatfA* strain was also sensitive to heat, oxidative (MSB, paraquat, *t*BOOH, H_2_O_2_), osmotic (1.2 M sorbitol), and cell wall (Congo Red, calcofluor white) stresses, as well as to iprodione, fluodioxonil, and caspofungin (0.5 to 1.0 μg/ml). It is noteworthy that *A. fumigatus* AtfA is also involved in the caspofungin paradoxical effect, i.e., in the elevated tolerance of high caspofungin concentrations (Valero et al. 2020). Some Atf transcription factors may also contribute to fine-tuning of the assimilation of carbon sources and the accumulation of reserves like trehalose and glycogen (Silva et al. 2021).

In *P. marneffei,* deletion of *atfA* increased the oxidative stress sensitivity of the fungus in the presence of *t*BOOH but its osmotic stress sensitivity and cell wall integrity remained unaltered (Nimmanee et al. 2014). Furthermore, inactivation of *atf1* resulted in higher sensitivity to heat stress (40 °C) in *Cryptococcus neoformans* yeast cells (Kim et al. 2010).

Extensive phenotypic characterizations of *atf1*-*atfA* orthologous gene deletion mutants have also been carried out in plant pathogenic fungi including *Fusarium* spp., *M. oryzae*, and *V. dahliae*. As expected, a number of stress sensitivity phenotypes have been recorded for the gene deletion strains, laying emphasis on the distinguished role of these bZIP-type transcription factors in the maintenance of the stress defense systems of these successful plant pathogens.

In *F. graminearum*, the *ΔFgatf1* deletion mutant showed increased sensitivity to oxidative (in the presence of 0.5 mM H_2_O_2_) and osmotic (tested with NaCl, KCl and sorbitol) as well as cell wall (150 μg/ml Congo Red treatment) stresses compared to the wild-type strain (Jiang et al. 2015; Nguyen et al. 2013). In *F. verticillioides*, *FvatfA* was also important in oxidative stress defense and cell wall integrity (Szabó et al. 2020). Deletion of *FvatfA* brought about increased sensitivity to H_2_O_2_, MSB, and CongoRed but heat and cold sensitivity of its conidia was comparable to that of the wild-type strain (Szabó et al. 2020).

In *M. oryzae*, both mycelia and conidia of the *Moatf1* deletion mutant were sensitive to H_2_O_2_ (Guo et al. 2010), and a high number of H_2_O_2_-treated conidia of the mutant were unable to germinate even after 12-h cultivation as compared with the wild-type strain (Guo et al. 2010). Interestingly, although altered intracellular accumulation of reactive oxygen species (ROS) was not detected, reduced laccase and peroxidase activities were measured in the *ΔMoatf1* strain, which is likely to increase the vulnerability of the fungus to the ROS-mediated defense of the host plant (Guo et al. 2010).

In *V. dahliae*, deletion of the *ATF* ortholog *VDAG_08676* gene caused no change in oxidative stress sensitivity of the fungus, but higher levels of ROS were generated in the mutant with concomitantly reduced laccase and peroxidase activities as compared to the wild type (Fang et al. 2017).

## Integration of Atf1-AtfA-FvAtfA orthologs into the fungal stress response regulatory network

AtfA and its orthologs are regulated by stress-activated protein kinases (SAPK) in fungi. In *S. pombe*, Atf1 is under the control of the well-known Sty1 SAPK which plays a predominant role in the regulation of genes during diverse forms of stress including heat, high osmolarity, oxidative, and starvation stresses (Kato Jr et al. 1996; Sánchez-Mir et al. 2018). Under various environmental stress conditions, Sty1 is phosphorylated, and then enters the nucleus where it binds and phosphorylates Atf1 leading to upregulation of several genes including the *atf1* gene itself (Gaits et al. 1998; Salat-Canela et al. 2017). It is also suggested that Atf1 is activated by the Pmk1 mitogen-activated protein kinase (MAPK) of the cell integrity pathway which is involved in the response to hypo- or hyper-osmotic stress, oxidative stress, glucose starvation stress, or cell wall damage stress, and contributes to cytokinesis and the maintenance of ion homeostasis (Takada et al. 2007; Pérez et al. 2018). Atf1 is constitutively localized in the nucleus under unstressed and nitrosative stress conditions; however, upon prolonged stress, it translocates into the cytoplasm for unknown reasons (Kar et al. 2018). Atf1 upregulates most of its target genes as a heterodimer formed with Pcr1. A few genes, however, do not need Pcr1 for their upregulation (Sansó et al. 2008). These genes may be upregulated by an Atf1 homodimer or by a not yet identified heterodimer of Atf1 (Sansó et al. 2008). As an example, Atf21 is postulated as a potential partner for heterodimer formation with Atf1 under KCl-induced stress (Sansó et al. 2008). Atf1 can interact with other transcription factors on the promoters of its target genes as well: Salat-Canela et al. (2017) suggested that Atf1 and Pap1 act synergistically during the upregulation of antioxidant enzyme genes and both transcription factors should be loaded on DNA for full upregulation. Moreover, due to its chromatin remodeling activity, Atf1 downregulates the basal expression of several genes under unstressed conditions (Sansó et al. 2011). Apart from its DNA binding function, Atf1 can physically bind to the anaphase-promoting complex/cyclosome (APC/C) as well and this protein-protein interaction is important in controlling cell cycle progression (Ors et al. 2009).

In *A. nidulans* and *A. fumigatus*, both activity of the AtfA protein and transcription of the *atfA* gene is controlled by the SakA (HogA) SAPK pathway under stress (Hagiwara et al. 2009; Miskei et al. 2009; Silva et al. 2017). When phosphorylated, SakA binds to AtfA in the nuclei and this step is necessary for AtfA-dependent gene activations in both species (Lara-Rojas et al. 2011; Silva et al. 2021). SakA together with AtfA contributes to oxidative, osmotic, and nutrient starvation stress responses and they are required for the proper regulation of asexual and sexual development in *A. nidulans* (Kawasaki et al. 2002; Lara-Rojas et al. 2011). In *A. fumigatus*, SakA and AtfA are important in adaptations to oxidative, osmotic, cell wall damage, and heat stresses (de Oliveira Bruder Nascimento et al. 2016). Manfiolli et al. (2019) demonstrated in *A. fumigatus* that SakA physically interacts with other MAPKs like the functionally redundant MpkC (de Oliveira Bruder Nascimento et al. 2016), a paralog of SakA, or MpkA (Valiante et al. 2015), a key player in the maintenance of cell wall integrity. These observations suggest that like in *S. pombe* (Takada et al. 2007; Pérez et al. 2018), AtfA is under the control of more than one MAPKs/SAPKs in *Aspergillus* species. AtfA was constitutively nuclear localized under unstressed conditions and upon stress in *A. fumigatus,* too (Silva et al. 2021). It contrasts with the behavior of AtfB, AtfC, and AtfD bZIP transcription factors of the same species, where the distribution between cytosol and nucleus highly depends on stress conditions, and their pronounced nuclear location was only observed under stress (Silva et al. 2021). Physical interaction between AtfA and AtfB was first suggested by Lara-Rojas et al. (2011) in *A. nidulans* and, more recently, it was demonstrated that besides the genetic interaction with the AtfA-D transcription factors in *A. fumigatus*, AtfA was also able to form heterodimers with all the other three tested bZIP transcription factors under various stress conditions (Silva et al. 2021).

Transcriptome data demonstrated that, like in the case of *S. pombe* Atf1, *A. nidulans* AtfA also regulated the basal transcription of many stress-related and stress-unrelated genes in unstressed cultures (Emri et al. 2015; Orosz et al. 2017). Genome-wide transcriptional changes detected concomitantly in a wild-type and an *atfA* gene deletion mutant suggested that besides its direct effects, AtfA also regulated the transcription of numerous genes indirectly during stress treatments (Orosz et al. 2017; Antal et al. 2020). It was postulated that AtfA was involved in the transcriptional upregulation of two component signal transduction system genes (including *phkA*, *phkB*, *fphA*, *ypdA*, *tcsB*) in *A. nidulans* based on their systemic downregulation in the *atfA* gene deletion mutant under versatile culturing conditions and the occurrence of putative Atf-binding sites in their promoters (Antal et al. 2020; Emri et al. 2021). Interestingly, some of the related proteins like NikA, YpdA, SskA, TcsA, and TcsB are upstream regulators of SakA in this fungus (Hagiwara et al. 2009; Miskei et al. 2009; Lara-Rojas et al. 2011). This postulated regulatory function of AtfA could modulate the activity of the signaling network upon stress leading to indirect transcriptional changes of a huge number of genes (Antal et al. 2020; Emri et al. 2021). Concurrently, deletion of *A. fumigatus sakA* encoding the upstream SAPK regulator of AtfA prevented the upregulation of two component signal transduction genes under stress (Silva et al. 2017).

Nevertheless, further studies are definitely needed and advisable (i) to describe the whole spectrum of genes under direct AtfA control, (ii) to shed light on the interacting partners of AtfA forming, e.g., heterodimers with it, and (iii) to reveal the interplays and cross-talks existing between AtfA and other stress response, developmental, and vegetative growth maintenance regulatory elements.

Summing it up, recent data suggest that the regulatory role of Atf1-AtfA-FvAtfA orthologs in fungi goes far beyond the upregulation of certain genes when it is necessary. They also affect the basal transcription level of genes under unstressed conditions and modulate the activity of the signaling network via physical interactions with other transcription factors and the transcriptional regulation of the signaling genes themselves upon stress.

## Atf1-AtfA-FvAtfA orthologs are important players in the regulation of secondary metabolism

A growing body of experimental data indicates that the biosynthesis of secondary metabolites (SMs) is tightly coupled to the oxidative stress response of fungi. AtfB, another bZIP transcription factor, together with additional transcription factors, including AP-1, MsnA, SrrA, and possibly AtfA were found to regulate both aflatoxin biosynthesis genes and oxidative stress response genes in *Aspergillus parasiticus* (Hong et al. 2013a). Based on the co-regulation of SM biosynthetic and oxidative stress response genes found in a number of fungi, a theory of double defense system working against ROS-elicited damages has been proposed by several researchers (Roze et al. 2011; Yin et al. 2012; Hong et al. 2013b). In this defense system, antioxidant scavengers constitute the first line, while production of SMs forms the second line of defense. The prominent role of Atf1-AtfA-FvAtfA orthologs in response to oxidative stress as proven in a variety of fungus species indicates that these transcription factors may also be important players in the regulation of SM biosynthesis (Emri et al. 2015; Jung et al. 2016; Pérez-Arques et al. 2019; Jung et al. 2016; Nguyen et al. 2013; Temme et al. 2012; Szabó et al. 2020). However, studies on the possible direct role of Atf1-AtfA-FvAtfA-type transcription factors in the regulation of SM production, i.e., when SM producing capabilities of the appropriate gene deletion mutants and their wild-type parental strains were compared in vitro, gave diverse results (Emri et al. 2015; Jung et al. 2016; Pérez-Arques et al. 2019; Jung et al. 2016; Nguyen et al. 2013; Temme et al. 2012; Szabó et al. 2020).

Comparing the global gene expression profiles of an *A. nidulans ΔatfA* mutant, characterized by an elevated sensitivity to oxidative (H_2_O_2_, MSB, tBOOH, diamide) stress with profiles of its parental wild-type strain, revealed remarkable transcriptional differences under either unstressed or stressed conditions (Emri et al. 2015). In the absence of stress, 657 and 542 genes were upregulated and downregulated, respectively, in the mutant in comparison to the wild-type strain. In subsequent stress exposure experiments, two levels of stress conditions were investigated: treatments with MSB, tBOOH, and diamide generated “severe” stress, while H_2_O_2_ and NaCl treatments caused “mild” stress. As expected, more than 1000 genes were differentially regulated in cultures subjected to “severe” stress, while “mild” stress treatments resulted in less profound global transcriptional changes (Emri et al. 2015). Importantly, stress exposures also altered the transcription of a number of putative SM genes; altogether, 155 and 112 SM genes were upregulated and downregulated, respectively, in the wild-type reference strain. On the other hand, the same stress treatments resulted in significantly enhanced gene expression changes in the *ΔatfA* mutant, where 179 and 139 SM genes were upregulated and downregulated, respectively (Table 2) (Emri et al. 2015). These findings clearly demonstrated that all kinds of oxidative stressors significantly affected the transcription of SM biosynthesis genes and, most importantly, AtfA turned out to be a key player in these events.

**Table 2:** Secondary metabolite gene clusters affected by *atfA* gene deletion in *Aspergillus nidulans*

Cluster (number of genes in the cluster^a^)	Stress dependence (number of stress-responsive genes in the cluster)
Clusters become stress responsive in the absence of AtfA in *A. nidulans*	
Monodictyphenone (mdp) cluster (12)^b^	Upregulated in H_2_O_2_ stress (11)
	Downregulated in *t*BOOH stress (8)
	Downregulated in NaCl stress (8)
Derivative of benzaldehyde1 (dba) and F9775 hybrid cluster 1 (9)^b^	Upregulated in H_2_O_2_ stress (5)
	Downregulated in *t*BOOH stress (6)
	Downregulated in diamide stress (6)
	Downregulated in NaCl stress (6)
pkf cluster (6)^b^	Upregulated in H_2_O_2_ stress (4)
	Downregulated in *t*BOOH stress (4)
	Downregulated in diamide stress (5)
	Downregulated in NaCl stress (5)
ivo cluster (2)^b,c^	Upregulated in H_2_O_2_ stress (2)
	Downregulated in *t*BOOH stress (2)
	Downregulated in diamide stress (2)
	Downregulated in NaCl stress (2)
AN7884 cluster (15)^d^	Upregulated in CdCl_2_ stress (7)
AN7084 cluster (9)^d^	Upregulated in CdCl_2_ stress (6)
Clusters lost their stress responsiveness in the absence of AtfA in *A. nidulans*	
Derivative of benzaldehyde1 (dba) and F9775 hybrid cluster 2 (10)^b^	Upregulated in *t*BOOH (6)
AN1242 cluster (6)^d^	Downregulated in CdCl_2_ stress (4)

^a^Only genes of clusters determined by either manually or experimentally were involved in the analysis (Inglis et al. 2013)

^b^Only clusters where more than the half of the cluster genes including at least one key gene showing upregulated or downregulated were regarded as stress-responsive cluster. In this case, genes showing at least two-time increase or decrease in their relative transcription levels were regarded as upregulated and downregulated genes, respectively (Emri et al. 2015)

^c^Out of the clusters containing four or less genes only the cluster where all the cluster genes showed upregulated or downregulated were regarded as stress-responsive cluster

^d^Only the clusters where the cluster genes were significantly enriched (Fisher’s exact test, *p* <0.05) in the upregulated or downregulated gene sets were regarded as stress-responsive cluster. In this case, all differentially expressed genes were regarded as stress-responsive genes (Emri et al. 2021)

In addition to oxidative stress, other types of environmental stresses, e.g., osmotic (NaCl) and heavy metal (Cd^2+^) stress, may also affect significantly the expression patterns of SM biosynthesis gene clusters in *A. nidulans* (Table 2). Importantly, H_2_O_2_ and Cd^2+^ exposures seem to be effective in the upregulation of various SM clusters in the *ΔatfA* mutant (Table 2). This observation may lead to the development of novel strain improvement strategies and even new industrial SM production platforms. Nevertheless, other kinds of stress treatments (*t*BOOH, diamide, NaCl) turned out to be mostly repressive for many SM clusters in the absence of *atfA* and the loss of AtfA even resulted in reduced stress responsiveness for some clusters (Table 2). These findings clearly indicate the limits of this approach (deletion of *atfA* combined with stress treatments) to activate inactive SM biosynthetic gene clusters in the Aspergilli (Emri et al. 2015; Reverberi et al. 2008, 2012; Amare and Keller 2014).

Atf1-AtfA orthologs may play an important role in the regulation of SM biosynthesis genes in human pathogenic fungi, too. For example, the *A. fumigatus* GprK G protein-coupled receptor, which stimulates the transcription of *atfA* and *sakA*, was also involved in the regulation of oxidative stress response and gliotoxin biosynthesis (Jung et al. 2016). It is important to note that gliotoxin, secreted by *A. fumigatus* conidia, is a prerequisite of the successful invasion of host by decreasing ciliary movement of bronchial epithelial cells (Tomee and Kauffman 2000). In *M. circinelloides*, a *Δatf1* mutant and its wild-type parental strain were co-cultured with mouse macrophages to evaluate the behavior of the mutant in the stressful environment present during phagocytosis (Pérez-Arques et al. 2019). In vitro host–pathogen RNA sequencing was then performed to assess the transcriptional differences between the two strains. Altogether, 3384 genes were differentially expressed in the *Δatf1* mutant in comparison to the wild-type strain, among which 15 were annotated as putative SM biosynthesis genes and their vast majority (14 out of 15) were downregulated in the absence of functional Atf1.

The regulatory function of Atf1-AtfA-FvAtfA orthologs on SM production is especially well studied in some plant pathogenic fungi. For example, *ΔFgatf1* mutants constructed in *F. graminearum* strain PH-1 by Nguyen et al. (2013) showed elevated tolerance to hydrogen peroxide when tested on a complete medium (CM) supplemented with 10, 15, and 20 mM H_2_O_2_ and they also produced some 3.5 and 1.7 times more deoxynivalenol (DON), a trichothecene mycotoxin after 1 and 3 days’ culturing in vitro, respectively, as compared to the wild-type strain. The increased DON production paralleled with a significant upregulation of key trichothecene biosynthesis genes (*tri4*, *tri5*, *tri6*, and *tri10*). Importantly, DON production of the *ΔFgatf1* strain was strongly reduced during infection of wheat heads. The *ΔFgatf1* strains that also produced higher amounts of aurofusarin, based on the intense red color of their colonies and genes (*gip1*, *gip2*, *pks12*) involved in aurofusarin biosynthesis, were upregulated in the mutant. On the other hand, production of zearalenone (ZEA), a third important SM of *F. graminareum* as measured under in vitro conditions, was similar in the gene deletion mutants and the wild-type and transcript levels of ZEA biosynthesis genes (*zea1*, *zeb1*, *zeb2*) were also similar in the mutants and their parental strain.

Studies using another *ΔFgatf1* mutant of *F. graminearum* PH-1 gave somewhat different results suggesting that the regulatory effect of FgAtf1 may diverge even at inter-strain level and the results greatly depend on experimental conditions. The gene deletion mutant of this fungus, produced by Jiang et al. (2015) showed slightly reduced growth on CM supplemented with 0.5 mM H_2_O_2_ as compared with the wild type indicating that the sensitivity of this fungus towards ROS increased in the absence of functional Atf1. Furthermore, no significant differences were observed in DON production, tested in rice grain cultures between the mutant and its parental strain suggesting that deletion of *atf1* has no influence on trichothecene biosynthesis.

In *F. verticillioides,* deletion of *FvatfA* led to dramatic changes in SM production. Concentrations of the polyketide-type mycotoxins, fumonisin B1 and B2 (FB1, FB2), potential causes of life-threatening disorders in humans and animals upon ingestion were below the detection limit as measured by CE-MS in the gene deletion mutant grown in Myro medium for 14 days, while the wild type produced normal amounts of these metabolites under the same conditions. Expression of two fumonisin biosynthesis genes, *fum1* and *fum8*, paralleled with fumonisin production: transcript levels of these two genes were significantly lower in the *ΔFvatfA* mutant in comparison to the wild type. Furthermore, the gene deletion mutant produced only trace amounts of carotenoids when cultured in liquid minimal medium under continuous illumination for 7 days, whereas the wild-type synthesized normal amounts of these SMs. *CarRA* and *CarB*, two key enzyme genes of carotenoid biosynthesis, were significantly downregulated in *ΔFvatfA.* In silico, promoter analyses aimed to trace the presence of the consensus sequence, TGACGTCA (known as the binding site for ATF/CREB bZIP transcription factors), on promoters of the genes found to be downregulated in the *ΔFvatfA* mutant which led to the identification of this motif on the sequences of *fum1*, *fum8*, *carRA*, and *carB*. On the other hand, the *ΔFvatfA* mutant produced approximately 10 times more bikaverin, a deep-red pigment with antimicrobial and anticancer activities than the wild type. Surprisingly, expression of *bik1*, a key polyketide synthase gene, responsible for bikaverin synthesis in *F. verticillioides* showed no increase in the mutant indicating that the causes of overproduction of this metabolite are others than upregulation of *bik1*. The most likely explanation of bikaverin overproduction is the drastic downregulation of the fumonisin and carotenoid pathways in *ΔFvatfA*. Due to these changes in the mutant, the building blocks needed for the production of fumonisins and carotenoids were probably accumulated and channelled towards the synthesis of other metabolites, including bikaverin. An interesting novelty of this research was the strongly reduced carotenoid production observed in the absence of AtfA. As mentioned before, SMs have been suggested to play a role in protection against ROS, but direct proofs of ROS-scavenging ability of most of these metabolites are lacking. Carotenoids are, however, well-documented antioxidants (Avalos and Limón 2015); their regulation by such a key player of stress mediation, like Atf1/AtfA, provides an unambiguous proof on the causal relation between ROS stress and response by SM production (Szabó et al. 2020).

Although deletion of *atf1* caused no change in oxidative stress sensitivity of *B. cinerea*, the *ΔBcatf1* mutant was found to produce higher amounts of secondary metabolites (botrydial, botryendial, and botcinin) as compared to the wild type and a number of genes coding for botrydial and botcinic acid was also upregulated in the mutant (Temme et al. 2012). Studies on the genome-wide effects of BcAtf1 brought to light the exceptional importance of this TF on SM production. When total mRNA samples of *B. cinerea* wild type and its *ΔBcatf1* mutant grown under unstressed conditions were subjected to microarray analyses, 511 genes including 34 predicted SM-encoding ORFs were found to be differentially expressed in the mutant (Temme et al. 2012). The number of SM genes, upregulated in the mutant, was significantly higher (26) than the number of downregulated ones (8) indicating that Atf1 exerts a repressing effect on the majority of SM genes at least in this fungus and under culture conditions allowing optimum growth.

In summary, studies on the effect of the deletion of *atf1-atfA-FvatfA* orthologous genes on SM production gave contrasting results: loss of this bZIP-type transcription factor had either positive or negative influence on SM production, depending on the studied species and the biosynthesis route of the given SM molecule. However, in basically ROS-sensitive fungus species, like *F. verticillioides*, inactivation of *FvatfA* resulted in a significantly elevated sensitivity to ROS and this effect was accompanied with strongly retarded production of SMs, including compounds (e.g., carotenoids) with proven antioxidant activity.

The role of Atf1-AtfA-FvAtfA-type transcription factors in regulating SM production in fungi may have a great biotechnological importance. Disarmed, mycotoxin non-producing strains can be constructed for use in food and fodder industry or in biological control by gene disruption or RNAi-mediated gene-silencing technologies (Szabó et al. 2020; Damann 2014; Kagot et al. 2019). Furthermore, the manipulation of *atf1-atfA-FvatfA* expression may cause dramatic changes in the SM spectra of various industrial microorganisms and, therefore targeted gene expression changes may result in overproduction of highly valuable metabolites, like carotenoids or bikaverin (Szabó et al. 2020; Thomas et al. 2017).

## Atf1-AtfA-FvAtfA orthologs affect virulence in pathogenic fungi

Functional analyses of *atf1/atfA* orthologs have been carried out in several human pathogenic fungus species, all of them classified as opportunistic pathogens.

In *A. fumigatus*, causing life-threatening lung and systemic infections in vulnerable, mostly immunocompromised patients, the abundantly disseminated small, stress-tolerant conidia with hydrophobic surface serve for efficient infection sources. AtfA was found to be essential for maturation of conidia and maintaining them in metabolically inactive, stress-tolerant dormancy stage, ready for dissemination and infection (Hagiwara et al. 2014, 2016); therefore, this transcription factor can be regarded as a basic component of virulence in this fungus. In another study, Silva et al. (2017) confirmed that AtfA is an important virulence factor of *A. fumigatus* using *Galleria mellonella* and neutropenic murine model of invasive pulmonary aspergillosis. Mortality rates of the *ΔatfA* mutant in *G. mellonella* were significantly lower as compared to the wild type strain. In the neutropenic murine model, the *ΔatfA* mutant also showed decreased virulence accompanied with weakened fungal growth (Silva et al. 2017) (Table 3).

**Table 3:** Atf1-AtfA-FvAtfA orthologs involved in the virulence of fungal species

Species	Orthologs	Mechanism of action	Reference
*A. fumigatus*	AtfA	Essential for maturation of conidia Increases survival in neutropenic murine model and in *Galleria mellonella*	Hagiwara et al. 2014, 2016; Silva et al. 2017
*M. circinelloides*	Atf1	Supports survival of spores in phagocytes Promotes germination of spores in phagosome	Pérez-Arques et al. 2019
*P. marneffei*	AtfA	Increases survival of conidia in mouse and macrophages	Nimmanee et al. 2014
*C. neoformans*	Atf1	Induces the thioredoxin antioxidant system	Missall and Lodge 2005
*F. oxysporum* f. sp. *cubense*	FoAtf1	Regulates ROS-scavenging enzymes	Qi et al. 2013
*F. verticillioides*	FvAtfA	Increases invasive growth tested on tomato fruits	Szabó et al. 2020
*M. oryzae*	MoAtf1	Contributes to the growth of infectious hyphae Induces laccase and peroxidase enzymes	Guo et al. 2010
*C. purpurea*	CPTF1	Contributes to normal catalase activity	Nathues et al. 2004
*B. cinerea*	BcAtf1	Involved in conidium production	Temme et al. 2012
*F. graminearum*	FgAtf1	Positively regulates DON production	Nguyen et al. 2013

*M. circinelloides* is an emerging pathogen infecting mainly immunocompromised individuals often with a fatal outcome. In this fungus, Atf1 (together with Atf2) plays a key role in virulence by supporting survival of the spores after internalization by phagocytes and promoting spore germination within the phagosome (Pérez-Arques et al. 2019). The two transcription factor-encoding genes were strongly induced during macrophage phagocytosis of the wild-type fungus. Deletion of either *atf1* or *atf2* resulted in drastic reduction of virulence as tested on immunosuppressed mice. Transcriptomic experiments, performed in a mouse model, where mouse macrophages and *Mucor* spores were co-cultured for five h and then subjected to RNA sequencing, demonstrated that Atf1 and Atf2 regulate the expression of a large bulk of target genes in the intraphagosomal environment: 3384 and 3644 genes were differentially expressed in *Δatf1* and *Δatf2*, respectively, as compared to their wild-type parent. (More than one-third of these genes were similarly regulated by the two transcription factors, suggesting an overlap between their functions.). Transcriptomic analyses revealed that Atf1 (and Atf2), besides regulating oxidative stress response, induces a variety of cellular processes including translation, carbon, and nitrogen metabolism, carotenoid biosynthesis, and cell wall biogenesis strengthening thus vigor, adaptation capability and virulence of the fungus (Table 3).

Deletion of *atfA* led to reduced virulence in *P. marneffei*, the cause of serious, often fatal systemic diseases in immunocompromised patients (Nimmanee et al. 2014). When mouse (J774) and human (THP1) macrophages were infected with conidial suspensions, survival of the *ΔatfA* mutant evaluated after 24-h incubation was found to be reduced by nearly 50% as compared to the wild type. The possible reasons of this reduced virulence remained unsolved, as the in vitro assays performed on artificial medium to determine sensitivity of the mutant to oxidative stressors gave diverse results: while the *ΔatfA* mutant was more sensitive to *t*BOOH, it showed no elevated sensitivity to hydrogen peroxide and menadione comparing with the wild type. The authors (Nimmanee et al. 2014) concluded that *atfA* is definitely required for virulence in *P. marneffei*, but its major role in such a complex trait is other than mediating the oxidative stress response of this fungus (Table 3).

*C. neoformans* is a basidiomycetous yeast-causing lung infection, meningitis, and encephalitis mainly as a secondary invader of AIDS patients. (Kim et al. 2010). Pioneer studies on an *atf1* ortholog of *C. neoformans* pointed out that this transcription factor-encoding gene may affect virulence, at least indirectly by regulating important elements of the oxidative stress tolerance system (Missall and Lodge 2005). Deletion of *atf1* resulted in elevated sensitivity to oxidative stress of the *Δatf1* strain, but the mutant displayed not much loss of virulence, as its survival rate in macrophages was similar to that of the wild-type parental strain in a mouse inhalation model. However, the thioredoxin antioxidant system of the gene deletion mutant suffered serious dysfunctions. The *trx1* gene showed delayed induction by oxidative stress and was significantly downregulated in a *Δatf1* background. Furthermore, the *Δtrx1* mutant had growth defects on artificial media and was sensitive to various stresses including oxidative stress. These findings indicate that Atf1 acts as an inducer of *trx1*, an indispensable element of the thioredoxin system in the presence of oxidative stress and protects by this way the fungus from ROS-mediated defense reaction of the host macrophages (Table 3).

As a summary of these studies, *atf1-atfA* seems to be indispensable for virulence in opportunistic human pathogens, but a causal relationship between ROS tolerance mediated by Atf1-AtfA and virulence was difficult to find. To overcome ROS produced by host immune cells is a prerequisite for these organisms to establish disease within animal/human microenvironments, but the absence or miss-function of *atf1-atfA* can be circumvented by the aid of other regulators, like AtfB/Atf2 or members of the Yap1 or Skn7 families that compete with, complement, or substitute for one another. Furthermore, *atf1-atfA* regulates other self-defense genes required for survival in the stressful animal/human environment as demonstrated elegantly by a recent study of Pérez-Arques et al. (2019).

Among the plant pathogenic fungi subjected to functional analysis of *atf1/atfA*, both hemibiotrophs/biotrophs and necrotrophs occur.

Deletion of *Foatf1* attenuated the virulence of *F. oxysporum* f.sp. *cubense*, responsible for the Panama disease of banana (*Musa* spp.). The gene deletion mutant of this fungus showed elevated sensitivity to H_2_O_2_ and reduced catalase activity, together with downregulation of catalase-encoding genes (Qi et al. 2013) confirming that *atf1* is requested for virulence in biotrophic and hemibiotrophic fungi due to its regulatory role on genes encoding ROS-scavenging enzymes, like laccases, catalases, and peroxidases needed by the pathogens to impair the plant defense, mediated by reactive oxygen species (Table 3).

Another example of biotrophic/hemibiotrophic fungi subjected to functional analysis of the *FvatfA* gene is the maize pathogen *F. verticillioides*. A *ΔFvatfA* mutant of this fungus also possessed increased sensitivity to oxidative stress-generating agents and its invasive growth, tested on tomato fruits, was significantly reduced in comparison to the wild type (Szabó et al. 2020) (Table 3).

Inefficient ROS scavenging was also found to play an important role in loss of virulence, when *Moatf1*, the *atf1* orthologous gene of of the rice blast fungus, *M. oryzae*, was investigated. The *ΔMoatf1* gene deletion mutant cultivated on artificial medium had increased sensitivity to H_2_O_2_ and displayed markedly reduced virulence on rice plants, fully susceptible to the wild-type parental strain. Infectious hyphae of the mutant showed retarded growth, accompanied with aberrant morphological changes (Table 3). Strong defense responses, including H_2_O_2_ accumulation around appressoria of the fungus, were induced by the mutant at the infection site. However, when H_2_O_2_ generation was prevented by treating the host leaf sheaths prior to fungal inoculation with diphenyleneiodonium, a potent inhibitor of NADPH oxidase, the major source of H_2_O_2_ production, normal infectious growth was restored in the mutant. Transcript levels of defense-related genes of both the jasmonic acid and the salicylic acid pathways (*Lox*, *PBZ1*, *AOS2* and *PR1a*, *PAD4*, *Cht1*, respectively), as measured by qRT-PCR, were much higher in plants infected with the mutant in comparison to the wild type-infected plants. The major cause of impaired virulence of the *ΔMoatf1* mutant was the downregulation of laccase and peroxidase-encoding genes paralleled with a strongly reduced levels of the ROS-detoxifying enzymes encoded by these genes (Guo et al. 2010) (Table 3).

Studies on *C. purpurea*, a biotrophic pathogen of grasses and small grain cereals, demonstrated that the ATF1 homolog, CPTF1, is a general regulator of catalase activity. The *Δcptf1* deletion mutants showed significantly weakened virulence on rye as indicated by reduced growth rate of these fungi and retarded honeydew production by the test plants at the infection site. Moreover, the rye epidermal tissues infected with *Δcptf1*mutants reacted with a rapid oxidative burst, whereas such response was absent in tissues infected with the wild type (Nathues et al. 2004). According to these pioneer experiments, the most likely reason of the weakened virulence found for the gene deletion mutants was their greatly impaired catalase activity. In the absence of normal catalase activity, these fungi were unable to scavenge H_2_O_2_ produced by the plant as a defense reaction, consequently they suffered serious ROS damage; furthermore, insufficiently scavenged H_2_O_2_, acting as a second messenger could efficiently alarm PTI (pathogen-triggered immunity) in the infected tissues (Table 3).

Contrarily to the situation found for biotrophic fungi, deletion of *atf1-atfA-FvatfA* had not much influence on ROS sensitivity in necrotrophic phytopathogens and the causes of altered virulence of the *Δatf1-ΔatfA-ΔFvatfA* mutants of necrotrophs, if happened at all were others than ROS tolerance.

Deletion of the *atf1* orthologous *Bcatf1* gene in *B. cinerea*, a necrotrophic fungus with wide host range resulted in no significant change of oxidative stress sensitivity comparing with the wild type. The *ΔBcatf1* mutant grew extremely vigorously and its colonization efficiency assessed on bean leaves and cucumber fruits was much stronger than that of the wild type (Temme et al. 2012). The aggressive colonization capability of the *ΔBcatf1* strain was of course insufficient to reach real hypervirulence, as this mutant was hampered in conidium production required for completing the disease cycle under natural conditions (Table 3). The improved colonization efficiency of *ΔBcatf1* was most probably due to its profound mycelial growth which appeared even on artificial medium, as well as the enhanced production of phytotoxins, including botrydial, botryendial, and botcinin.

*ΔFgatf1* mutants of *F. graminearum*, PH-1 a mycotoxin-producing pathogen of maize and small grain cereals, were less sensitive to oxidative stress compared with the wild type, but they showed strongly reduced virulence on wheat and weakened colonization efficiency on maize cobs. Constitutive expression of *Fgatf1* resulted in hypervirulence on *Brachypodium distachyon*, maize and wheat, while the *Fgatf1*^*oe*^ strain remained as sensitive to oxidative stress as the wild type suggesting that *atf1* can enhance virulence without the improvement of ROS tolerance in this necrotrophic fungus. The *ΔFgatf1* mutants produced less DON *in planta* by day 7 after infection than the wild type (Nguyen et al. 2013), and therefore the impaired mycotoxin production detected within plant tissues could be a reason of the attenuated virulence found for the gene deletion strains, since DON is a virulence factor due to its inhibitory effect on protein synthesis (Table 3). However, the possible role of other, still unknown factors, downregulated in the *Δatf1* gene deletion mutant would be premature to neglect as a cause of reduced virulence. Jiang et al. (2015) studied functional relationships of three stress-related transcription factor genes, namely *Fgap1*, *Fgatf1*, and *Fgskn7* of *F. graminearum* PH-1. (*Skn7*, a conserved gene in filamentous fungi is required for the upregulation of a number of oxidative stress-responsive genes.) Both *ΔFgatf1* and *ΔFgsnk7* were only slightly more sensitive to H_2_O_2_ on complete medium than the wild type. (*NB.* This *ΔFgatf1* strain was different from the mutants studied by Nguyen et al. (2013).). When flowering wheat heads were artificially infected with these fungi, the *ΔFgatf1* mutant displayed nearly fourfold reduced disease index as compared to the wild type, whereas the *ΔFgsnk7* mutant was as virulent as the wild type indicating again that deletion of *atf1* leads to attenuated virulence without significantly altered H_2_O_2_ sensitivity. DON production of *ΔFgatf1* was similar to the wild type on sterilized rice grain, but in infected wheat heads the mutant produced less DON than the wild type. The *ΔFgsnk7* mutant produced much less DON on rice than the wild type, while its DON production in infected wheat heads was reduced only by 25% in comparison to the wild type. The *ΔFgsnk7–ΔFgatf1* double mutant was more severely hampered in DON production and its virulence was drastically reduced, indicating that (i) sufficient DON production rather than H_2_O_2_ insensitivity is an important virulence factor in *F. graminearum*, and (ii) an interplay is needed between Atf1 and other stress-related transcription factors to attain normal virulence in necrotrophic fungi.

The mode how *atf1/atfA/FvatfA* affects virulence is thus dependent on the lifestyle of plant pathogenic fungi. Biotrophs/hemibiotrophs require elevated ROS tolerance to achieve a successful pathogenic way of living and, therefore, Atf1-AtfA-FvAtfA, as an important element of the oxidative stress response, has a significant role in the regulation of their virulence. Contrarily, nectrotrophs need other factors for virulence (e.g., phytotoxin production, sporulation intensity, extracellular enzyme production) that may be regulated either positively or negatively by Atf1-AtfA-FvAtfA resulting in the diverse pathogenic behavior of *atf1/atfA/FvatfA* deletion mutants of these fungi. This conclusion is in harmony with a former paper of Heller and Tudzynski (2011), who pointed out that ROS tolerance was dispensable for virulence in necrotrophic plant pathogens.
